# The Characteristics of Mood Polarity, Temperament, and Suicide Risk in Adult ADHD

**DOI:** 10.3390/ijerph17082871

**Published:** 2020-04-21

**Authors:** Giancarlo Giupponi, Marco Innamorati, Elena Rogante, Salvatore Sarubbi, Denise Erbuto, Ignazio Maniscalco, Livia Sanna, Andreas Conca, David Lester, Maurizio Pompili

**Affiliations:** 1 Department of Psychiatry, Azienda Sanitaria dell’Alto Adige, 39100 Bolzano, Italy; giancarlo.giupponi@sabes.it (G.G.); maignazio@inwind.it (I.M.); liviasanna@gmail.com (L.S.); andreas.conca@asbz.it (A.C.); 2 Department of Human Sciences, Università Europea di Roma, 00163 Rome, Italy; marco.innamorati@unier.it; 3 Department of Psychology, Sapienza University of Rome, 00185 Rome, Italy; elena.rogante@uniroma1.it (E.R.); salvatore.sarubbi@uniroma1.it (S.S.); 4 Department of Neurosciences, Mental Health and Sensory Organs, Suicide Prevention Centre, Sant’Andrea Hospital, Sapienza University of Rome, 00189 Rome, Italy; denise.erbuto@gmail.com; 5 Department of Psychology, Stockton University, Galloway, NJ 08205, USA; david.lester@stockton.edu

**Keywords:** adult ADHD, suicide, affective temperaments, hypomania, Comorbidity

## Abstract

The present study was designed to shed light on a topic rarely explored and to suggest possible ways to detect risk factors for the presence of suicidal ideation and behaviors in a sample of adult patients with Attention-Deficit Hyperactivity Disorder (ADHD). This study also explored the association between ADHD, affective temperaments, the presence of hypomania symptoms, and suicide risk. We hypothesized that (compared to healthy controls) (1) patients with adult ADHD would report more negative affective temperaments and more hypomania symptoms and (2) that they would have a higher suicide risk. The participants included 63 consecutive adult inpatients (18 women, 45 men) with ADHD and 69 healthy controls (42 women, 22 men). All participants were administered the Wender Utah Rating Scale (WURS), the Hypomania Check-List-32 (HCL-32), the Mood Disorder Questionnaire (MDQ), the Temperament Evaluation for Memphis, Pisa, Paris, and San Diego (TEMPS-A), and the Columbia-Suicide Severity Rating Scale (C-SSRS). Forty-six percent of the ADHD patients had an Axis 1 comorbid disorder. ADHD patients (compared to controls) more often reported suicidal ideation (46.0% vs. 5.9%, one-way Fisher exact test *p* < 0.001; phi = 0.46). ADHD patients and the controls also significantly differed in all the scales administered (with Cohen’s d between 0.92–4.70), except for the TEMPS-A Hyperthymia scale. A regression model indicated that ADHD was independently associated with higher scores of a negative temperaments/hypomania factor (Odd Ratio = 14.60) but not with suicidal ideation. A high incidence of suicidal ideation, comorbid psychiatric disorders, and negative affective temperaments was reported in adult ADHD patients, and clinicians should routinely assess risk factors for suicide among these patients.

## 1. Introduction

Attention-Deficit Hyperactivity Disorder (ADHD) is a neurodevelopmental disorder with childhood-onset, marked by high and consistent levels of inattention, impulsivity, and/or hyperactivity that must be diagnosed before the age of 12 and can lead to functional impairment in multiple domains (familial, social, academic, and work-life) [1]. The predominance of symptoms varies between individuals and over time in the same individual, giving rise to different configurations of the disease: predominantly inattentive, predominantly hyperactive-impulsive, or a mixed clinical presentation of the two domains [2]. Corbisiero et al. [3] suggested that emotional dysregulation, consisting of higher irritability, affective lability, and emotional over-reactivity, and lower frustration tolerance, should be considered a core symptom of ADHD in children.

Although ADHD has long been conceptualized as a developmental disorder, it usually persists into adulthood. In fact, its global prevalence in the general adult population is around 2.8%, ranging between 0.6% and 7.3% [4,5]. Common symptoms in adults include restlessness, talkativeness, the inability to relax, excessive fidgeting, impatience, sensation-seeking behaviors, disorganization, distractibility, impaired decision-making, deficient emotional self-regulation, and sensitivity to stress [6,7]. It is also common to observe unhealthy habits, such as smoking, alcohol, and drug misuse [8], risky sexual behaviors, and altered sleep rhythms [9,10].

Higher healthcare utilization and costs for multimorbidity, especially for the inpatient care of psychiatric disorders, are generally reported among adults who have had childhood ADHD, remitted or not [11]. Many studies report the presence of comorbid psychiatric disorders in adults with ADHD [12,13,14] and also report ADHD in individuals with at least one psychiatric disorder [15]. A study carried out in an Italian sample of 634 psychiatric outpatients found a 6.9% prevalence of ADHD diagnosis [16].

The co-occurrence of ADHD and another psychiatric diagnosis can complicate the recognition of ADHD [17]. Furthermore, it has been suggested that early and optimal treatment of ADHD could potentially prevent the emergence of later psychiatric conditions. Mood disorders, anxiety, and personality disorders are the most common conditions associated with ADHD [18]. The rates of patients with ADHD and comorbid bipolar disorder have been estimated to be between 5.1–47.1%, with a higher prevalence of Bipolar I disorder than Bipolar II [19]. The specific features of ADHD patients with comorbid bipolar disorder, compared to other comorbidities, include chattiness, distractibility, restlessness, an earlier age of onset, a worse prognosis, shorter periods of wellness, and more frequent mood events [20]. The prevalence rates of depression among patients with Major Depression and comorbid ADHD range from 18.6% to 53.3%, with a lower self-reported quality of life than patients with Major Depressive Disorder alone [21]. The risk for anxiety disorder is higher among individuals with ADHD than for the general population, with rates near 50%, and ADHD is more prevalent in patients with social phobia than in those with panic disorder. The characteristics include more severe anxiety symptoms, an earlier onset, and more frequent comorbid psychiatric disorders. Moreover, in individuals with anxiety disorder, ADHD is diagnosed later [13].

Personality disorders, mainly those of cluster B and C, are estimated to be present in more than 50% of adults with ADHD [22], leading to greater impairment and lower response to treatment [23].

There is evidence of a positive association between ADHD and suicidality [24,25,26,27,28], especially in female patients [29]. For example, a large register-based population study from Sweden [30] revealed a greater increase in females than in males in the risk of suicide attempts among ADHD patients compared to the matched controls from the general population. Nigg et al. [25] found that women with ADHD are more likely to attempt suicide, while men are more likely to die by suicide.

The presence of psychiatric comorbidities could be another risk factor for suicide in patients with ADHD [28]. In a population-based study of the long-term outcomes of ADHD patients compared to the general population, Barbaresi et al. [26] found that the ADHD group had significantly higher suicide rates than the controls, with a standardized mortality ratio of 4.83 (95% CI; 1.14–20.46; *p* < 0.05).

The aim of the present study is to investigate the presence of suicidal ideation and behaviors in a sample of adult patients with ADHD and to explore the association between ADHD, affective temperaments, the presence of hypomania symptoms, and suicide risk. We hypothesized that (compared to healthy controls) (1) patients with adult ADHD would report more negative affective temperaments and more hypomania symptoms and (2) that they would have a higher suicide risk.

## 2. Materials and Methods

### 2.1. Study Participants

A total of 132 adults took part in the present study: 63 consecutive adult inpatients with ADHD were admitted to the study in the order of presentation (18 women, 45 men), according to the diagnostic criteria of the DSM-5 [1] from the General Psychiatry Clinic of Bolzano Hospital in 2017; 69 healthy controls (42 women, 22 men) were recruited among the students and healthcare professionals working at the hospital. The ethnic composition of the samples (Italian versus German) is shown in Table 1.

Inclusion criteria: All patients were between 18 and 65 years old, diagnosed with ADHD according to the diagnostic criteria of the DSM-5 [1], and admitted to the General Psychiatry Clinic at Bolzano Hospital. All controls were between 18 and 65 years old, with no psychiatric diagnoses. Exclusion criteria were major chronic diseases or deficits that prevented them from understanding and completing the evaluation.

The medical staff informed the participants about the relevance of the study, explaining its goals and purposes. All participants signed informed consent (Italian or German version) voluntarily. The Institutional Review Board approved the study (Number 12, 20/12/2016)

### 2.2. Measures

All the patients were administered the Wender Utah Rating Scale (WURS) [31,32], the Hypomania Check-List-32 (HCL-32) [33], the Mood Disorder Questionnaire (MDQ) [34], the Temperament Evaluation for Memphis, Pisa, Paris, and San Diego (TEMPS-A) [35,36], and the Columbia-Suicide Severity Rating Scale (C-SSRS) [37]. Sociodemographics (sex and age) and clinical information (DSM-5 diagnoses) were retrieved from medical records.

The Wender Utah Rating Scale (WURS) is a 61 item self-report instrument to measure the presence, frequency, and severity of ADHD symptoms experienced in childhood. Each item can be rated from 0–4 (0—not at all/very slightly; 1—mildly; 2—moderate; 3—quite a bit; 4—very much). Sample items include “As a child I experienced inattentive daydreaming” and “As a child in school I did not achieve my potential”. The score is calculated as the sum of the item answers, and a total score of 46 or more is consistent with a positive diagnosis for ADHD. For the present sample, Cronbach’s alpha was 0.98 for both the German and Italian samples.

The Hypomania Check-List-32 (HCL-32) is a self-report scale for the assessment of hypomanic symptoms. It has 32 items with yes/no answers that examine the emotions, behaviors, or thoughts linked to hypomania. The total score is calculated by adding up the affirmative answers, and a total score of 14 or more indicates that the subject is potentially bipolar. Sample items include “I feel more energetic and more active” and “I have more ideas; I am more creative”. For the present sample, the Cronbach’s alpha values were 0.91 for the Italian sample and 0.94 for the German sample.

The Mood Disorders Questionnaire (MDQ) is a self-report questionnaire featuring 13 items with yes/no answers to screen for hypomania or mania, including a final item measuring the level of impairment due to the symptoms on a 4-point scale. Sample items are “Has there ever been a period of time when you were not your usual self, and you were so irritable that you shouted at people or started fights or arguments?” and “Has there ever been a period of time when you were not your usual self and spent money or got you or your family into trouble?”. For the present sample, the Cronbach’s alpha values were 0.86 for the Italian sample and 0.89 for the German sample respectively.

The Temperament Evaluation Memphis for Pisa, Paris, and San Diego (TEMPS-A) is a self-report instrument to measure affective temperamental traits. It consists of 110 items with true/false answers, which result in 5-dimensional scales: Depressive, Cyclothymic, Hyperthymic, Irritable, and Anxious. Sample items are “I am the kind of person who does not like change very much” and “I am totally comfortable even with people I hardly know”. For the present sample, the Cronbach’s alpha values were 0.77 (Italian version) and 0.78 (the German version) for Depression; 0.91 for Cyclothymia (both for the Italian and German version); 0.79 (Italian version) and 0.83 (German version) for Hyperthymia; 0.87 (Italian version) and 0.86 (German version) for Irritability; and 0.87 (Italian version) and 0.89 (German version) for Anxiety.

The Columbia-Suicide Severity Rating Scale (C-SSRS) is a semi-structured interview used to assess suicidal ideation and suicidal behavior. The first part explores suicidal ideation (its intensity, frequency, duration, controllability, deterrents, and reasons). The second part of the interview examines suicidal behavior, including preparatory acts, interrupted or aborted attempts (the first type occurs when individuals start an action to end their lives but are stopped by someone or something external intervening, while the second type occurs when individuals stop themselves after attempting to end their lives), actual attempts, and non-suicidal self-injurious behavior.

### 2.3. Statistical Analyses

All analyses were performed with the statistical package for social sciences (IBM SPSS Statistics for Windows, Version 19.0, released 2010, Armonk, NY:IBM Corp ). One-way Fisher exact tests and chi-squared (χ^2^) tests were used for the 2 x 2 and N x N contingency tables, respectively. Independent sample t-tests were used to evaluate the differences between groups based on dimensional variables. A Bonferroni correction for multiple statistical tests was used to correct the *p* values in bivariate analyses. Phi (*φ*), Cramer’s *v* (*φ_c_*), and Cohen’s *d* (*d*) indices were reported as measures of the effect size. *φ* and *φ_c_* values of 0.20 and lower indicate a weak or negligible association, values between 0.20–0.40 show a moderate association, values between 0.40–0.60 indicate a relatively strong association, and values of 0.60 and higher indicate a strong association. *d* = 0.20 indicates a small effect size, *d* = 0.50 is a medium effect size, and *d* = 0.80 is a large effect size. Because of the high correlations between the negative affective temperaments, HCL-32 and MDQ scores, and potential problems with multicollinearity, we included these variables in a principal axis factor analysis and calculated the factor scores for use as independent variables in regression models. The odds ratios (ORs) and their 95% confidence intervals (CI) were reported as measures of associations. All tests were significant for *p* < 0.05.

## 3. Results

The ADHD patients and controls differed in sex (one-way Fisher exact test *p* < 0.001) and age but only before correction for multiple testing (t_130_ = −2.97, *p* = 0.004). The ADHD patients and controls did not differ in their ethnic composition (one-way Fisher exact test *p* = 0.50). The patients (compared to controls) were younger (36.78 ± 11.55 vs. 42.39 ± 10.19) and more likely to be male (71.4% vs. 31.9%).

### 3.1. Type of ADHD

Of the 63 ADHD patients, 15.9% presented with Hyperactive/impulsive symptoms, 11.1% with Inattentive symptoms, and 73.0% with a combination of Hyperactive/impulsive and Inattentive symptoms (Table 1). Furthermore, 46% of the ADHD patients had an Axis 1 comorbid disorder: 4.8% BD-1; 7.9% BD-2; 1.6% Cyclothymia; 12.7% Dysthymia; and 27.0% other disorders (e.g., PTSD, Substance abuse, Anxiety disorders).

### 3.2. Comorbid Psychiatric Disorder

The subgroups of ADHD patients did not differ in the presence of Axis 1 disorders. An Axis 1 disorder was present in 50.0% of the Hyperactive/impulsive group and 57.1% of the Inattentive group (χ^2^ = 0.09; df = 2; *p* = 0.95). A personality disorder was present in 20.0% of the Hyperactive/impulsive group and 28.6% of the Inattentive group (χ^2^ = 0.17; df = 2; *p* = 0.92). Suicidal ideation was present in 30.0% of the Hyperactive/impulsive group and 57.1% of the Inattentive group (χ^2^ = 1.44; df = 2; *p* = 0.49). A suicide attempt was present in 0.0% of the Inattentive group and 20.0% of the Hyperactive/impulsive group (χ^2^ = 2.05; df = 2; *p* = 0.36). Non-suicidal self-harm was present in 14.3% of the Inattentive group and 20.0% of the Hyperactive/impulsive group (χ^2^ = 0.16; df = 2; *p* = 0.93).

### 3.3. Suicidal Behaviors

ADHD patients (compared to controls) more often reported suicidal ideation (46.0% vs. 5.9%; one-way Fisher exact test *p* < 0.001), but only 4.8% of the ADHD patients reported active suicidal ideation with the intent to die and a specific plan. However, after correction for multiple testing, no between-group differences were statistically significant for suicidal behaviors. Only 9.5% of the ADHD patients had a history of an actual suicide attempt (vs. 0.0% of the controls; one-way Fisher exact test *p* = 0.020), 20.6% reported a history of preparatory acts or behaviors and interrupted attempts (vs. 0.0% of the controls), and 15.9% reported self-injurious behavior without suicidal intent (vs. 3.8% of the controls; one-way Fisher exact test *p* = 0.033). 

### 3.4. Temperament and Other Scale Scores

The ADHD patients and controls differed on all scales administered, except for TEMPS-A Hyperthymia (9.14 ± 4.09 vs. 10.14 ± 5.05; t130 = −1.25, *p* = 0.22) (Table 1). ADHD patients (compared to the controls) had higher scores for all negative affective temperaments, HCL-32, and MDQ.

Because of the high correlations between affective negative temperaments, the HCL-32 and MDQ scores, and the potential problems with multicollinearity, we included these variables in principal axis factoring, resulting in a single factor with an eigenvalue of 4.04 and 67.4% of the variance explained. The factor loadings ranging between 0.56 for the HCL-32 and 0.89 for TEMPS-A Anxiety). The factor score was included as an independent variable, along with sex, age, and suicidal ideation, in a logistic regression model with groups as the criterion. This model explained the group differences well (χ^2^ = 103.49; df = 4; *p* < 0.001; Nagelkerke R^2^ = 0.73). The ADHD patients (compared to the controls) were more likely to have higher scores in their negative temperament/hypomania factors (OR = 14.60; b = 2.68; SE = 0.53; *p* < 0.001). ADHD patients (compared to controls) were also more likely to be men (OR = 7.92; b = 2.07; SE = 0.62; *p* = 0.001). Age (OR = 0.96; b = −0.04; SE = 0.02; *p* = 0.10) and suicidal ideation (OR = 3.71; b = 1.31; SE = 0.85; *p* = 0.12) were not independently associated with the criteria.

## 4. Discussion

The present study was designed to investigate the differences in suicidal ideation, temperament, and manic and hypomanic symptoms between patients with adult ADHD and healthy controls. ADHD in adulthood, especially when concurrent with other mental diseases, may lead to major consequences for public health, such as higher healthcare costs and more difficult patient management [11].

In line with prior research findings [38], the descriptive statistics showed the prevalence of mixed ADHD clinical manifestation (Hyperactive/Impulsive and Inattentive combined) in our sample, and almost half of the patients presented with a psychiatric comorbidity. Several studies have investigated the presence of concomitant psychopathology among ADHD patients [39,40]. For example, Park et al. [39] conducted an epidemiologic study to analyze the prevalence and types of comorbid disorders in adult ADHD and determined that the most prevalent comorbidities were substance use, anxiety, and mood disorders. Given the high rate of comorbid psychiatric conditions, Soendergaard and colleagues [40] pointed out the importance of the prompt detection and treatment of both ADHD and psychiatric disorders to avoid complications in prognosis and treatment and to improve diagnostic precision.

Our results showed differences between adult ADHD patients and the healthy group regarding their age and sex, but these differences were not significant after correcting with multiple statistical tests. Past studies have sometimes indicated a higher prevalence of young males in ADHD samples [41,42]. The younger age could be a result of the developmental nature of ADHD, which has its onset in childhood and can persist into adulthood. Breda and colleagues [41] suggested that a biased recall of childhood symptoms, which are necessary to diagnose ADHD in adults, may affect the accuracy of the association with age. Moreover, the higher number of male ADHD patients is often associated with the greater externalization of symptoms among men [42].

The ADHD patients reported more frequent suicidal ideation than the healthy controls, and this result is consistent with prior investigations. Van Eck et al. [43] examined suicidal ideation in adults with ADHD and hypothesized that depressive symptoms can mediate this association, while emotional regulation can act as a moderator of this association. Furczyk and colleagues [28] suggested that the increased suicide risk among ADHD patients may be a result of psychiatric comorbidities and specific personality traits, such as impulsivity. However, in the present study, after controlling for multiple statistical tests, there were no between-group differences in suicidal behavior. This result may be due to the presence of psychiatric comorbidities, which are more powerful predictors of suicidal behavior than ADHD alone. Agosti et. underlined how the presence of comorbid conditions confers a four to twelve times greater risk of suicidal behavior [44].

The ADHD patients had higher scores than the controls on all scales of TEMPS-A (except for Hyperthymia), MDQ, and HCL-32. These results are consistent with previous research findings: Torrente et al. [45] reported that patients with ADHD had higher scores than controls for all negative affective temperaments (but not Hyperthymia), especially cyclothymia, followed by irritable temperament. The prevalence of these traits may confirm the hypothesis of emotional lability as a core symptom of ADHD [46]. Another study [47] underlined the differences in temperament between ADHD patients and healthy controls. Consistent with our results. In that study, all temperament scores, except for Hyperthymia, were higher among ADHD patients and patients with bipolar disorder.

Lastly, to detect possible risk factors for adult ADHD, we developed a logistic regression model with the clinical and control group as the criterion and the factor score for temperament as an independent variable, along with suicidal ideation, sex, and age. The results showed that being male and having higher scores for the temperament factor (including negative temperament, along with hypomania and mania symptoms) played a role in predicting the presence of ADHD in our sample. Age and suicidal ideation were not associated with ADHD. Landaas and colleagues [48] observed the prevalence of Cyclothymic temperament in adults with ADHD, and these patients also reported more symptoms of ADHD and mania or hypomania. It is interesting to note that cyclothymic temperament is the most commonly observed temperament among patients with bipolar disorder [49].

This study had some limitations. First, we assessed the mania/hypomania symptoms, ADHD symptoms, and affective temperaments through self-report questionnaires (MDQ; HCL-32; WURS; TEMPS-A), but we did not use direct observation of actual behaviors or any diagnostic interviews conducted by a clinician, except for suicidality. Observations and interviews may have been more reliable and allowed us to obtain more accurate and specific information about the patients’ mental health. Moreover, since all the questionnaires were self-reported, social desirability may have been present in the patients’ responses. Second, the number of participants was modest. Lastly, given the cross-sectional nature of the study, definitive conclusions cannot be made regarding the cause–effect relationships among the variables, and we did not consider fluctuations over time.

## 5. Conclusions

Clinicians should routinely assess the presence of comorbid psychiatric disorders and suicide risk in adult ADHD patients. Adult ADHD patients frequently report suicidal ideation, sometimes with specific plans to commit suicide. The size of the effect for history of suicide attempts and interrupted attempts was more limited. Negative affective temperament, hypomania, and deficient emotional self-regulation were also present, and their roles as risk factors for suicide should be investigated in future studies. The early detection of conditions that may worsen mental health or exacerbate further disorders is fundamental and could contribute to the design of more effective treatments. We recommend a multimodal approach that considers the different facets of ADHD and is effective in managing the social, professional, and emotional areas compromised by the disease.

## Figures and Tables

**Table 1:** Sociodemographic and clinical characteristics of the sample.

Variables	ADHD *N* = 63	Controls *N* = 69	Test	Significance	Effect Size
*Sex*				<0.001	0.40
Men	71.4%	31.9%			
Women	28.6%	68.1%			
Age	36.78 ± 11.55	42.39 ± 10.19	*t*_130_ = −2.97	0.004	−0.52
Ethnic group			-	0.50	0.02
Italian	60.3%	58.8%			
German	39.7%	41.2%			
Ladin	0.0%	0.0%			
Other	0.0%	0.0%			
ADHD group					-
None	0.0%	100.0%			
Combine	73.0%	0.0%			
Hyperactive/impulsive	15.9%	0.0%			
Inattentive	11.1%	0.0%			
DSM-V Axis 1 disorders	46.0%	0.0%			-
BD I	4.8%	0.0%			
BD II	7.9%	0.0%			
Cyclothymia	1.6%	0.0%			
Dysthymia	12.7%	0.0%			
Other specified disorders	27.0%	0.0%			
DSM-IV-TR Axis 2 personality disorders	23.8%	0.0%			
Suicidal ideation	46.0%	5.9%	-	<0.001	0.46
Death wishes	7.9%	1.5%			
Non-specific active suicidal thoughts	4.8%	0.0%			
Active suicidal ideation with any methods (not plan) without intent to act	15.9%	1.5%			
Active suicidal ideation with some intent to act, without specific plan	12.7%	2.9%			
Active suicidal ideation with specific plan and intent	4.8%	0.0%			
Preparatory acts or behavior	12.7%	0.0%		0.005	0.25
Aborted suicide attempt	0.0%	0.0%		-	-
Interrupted suicide attempt	7.9%	0.0%		0.037	0.20
Actual suicide attempt	9.5%	0.0%		0.020	0.22
Self-injurious behavior without suicidal intent	15.9%	3.8%		0.033	0.20
WURS	63.79 ± 12.14	12.83 ± 9.37	*t*_130_ = 27.14	<0.001	4.70
TEMPS-A Depression	11.35 ± 3.81	6.22 ± 2.65	*t*_130_ = 8.92	<0.001	1.56
TEMPS-A Cyclothymia	10.41 ± 5.51	3.70 ± 3.56	*t*_130_ = 8.23	<0.001	1.45
TEMPS-A Irritability	8.76 ± 4.50	3.16 ± 2.91	*t*_130_ = 8.40	<0.001	1.48
TEMPS-A Anxiety	13.53 ± 5.23	5.04 ± 3.87	*t*_130_ = 10.47	<0.001	1.85
TEMPS-A Hyperthymia	9.14 ± 4.09	10.14 ± 5.05	*t*_130_ = −1.25	0.22	−0.22
HCL-32	18.08 ± 7.27	11.55 ± 6.97	*t*_130_ = 5.27	<0.001	0.92
HCL-32 ≥ 14	68.3%	43.5%		0.003	0.25
MDQ	6.87 ± 3.53	2.61 ± 2.74			1.35

ADHD—Attention-Deficit Hyperactivity Disorder; DSM – Diagnostic and Statistical Manual of Mental Disorders; BD I – Bipolar disorder Type I; BD II – Bipolar disorder type II; WURS—Wender Utah Rating Scale; TEMPS-A—Temperament Evaluation Memphis, Pisa, Paris and San Diego; HCL-32—Hypomania Check-List-32; MDQ—Mood Disorders Questionnaire.
